# Supplementary figures and images for: Phylogeography of Aphyocypris normalis Nichols and Pope, 1927 at Hainan Island and adjacent areas based on mitochondrial DNA data
Source: PLoS One. 2023 Feb 28;18(2):e0282460. doi: 10.1371/journal.pone.0282460 (PMC9974131; doi:10.1371/journal.pone.0282460)

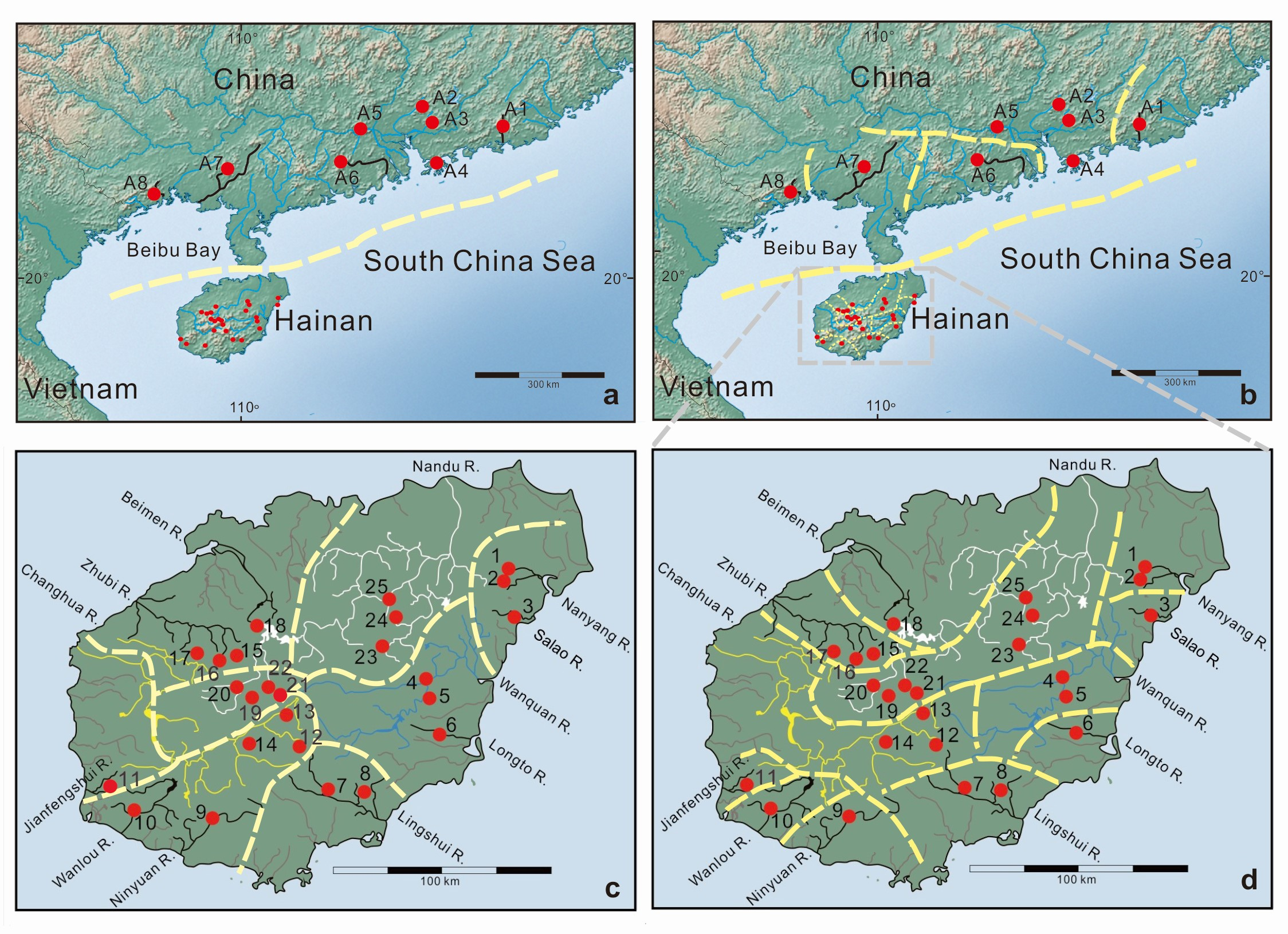

Supplement: S1 Fig — The four tested hypotheses via hierarchical analysis of molecular variance (AMOVA) in this study: a, isolated by the strait (hypothesis 1a); b, drainage by river system, both on Hainan Island and on the mainland (hypothesis 1b); c, eight biogeographical areas on Hainan Island modified from climate pattern [36] (hypothesis 2b); d, twelve groups model based on drainage basins on Hainan Island (hypothesis 2a). (TIF) [file pone.0282460.s002.tif]

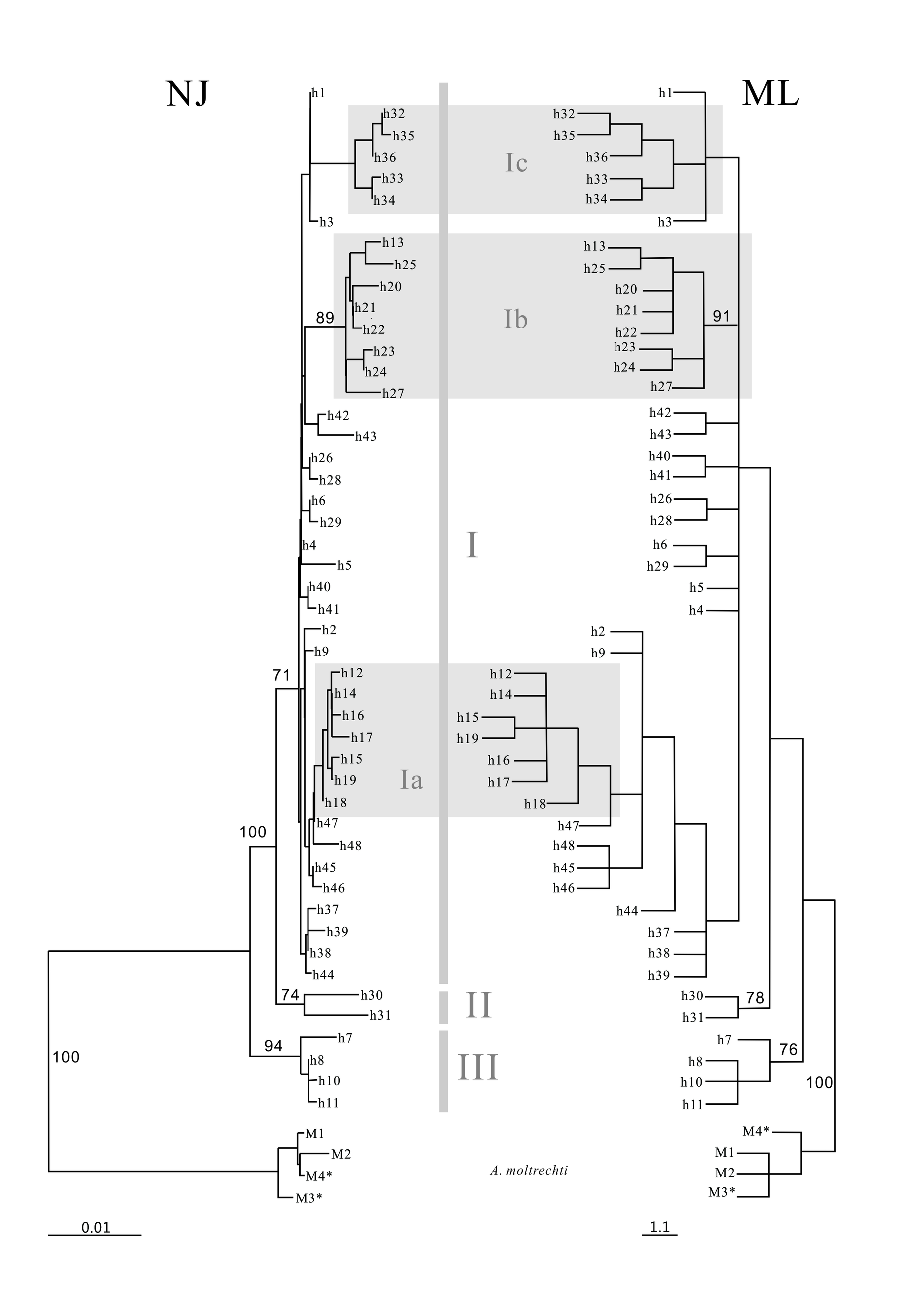

Supplement: S2 Fig — Numbers at internal nodes are bootstrap values and only value >70% are shown. (TIF) [file pone.0282460.s003.tif]

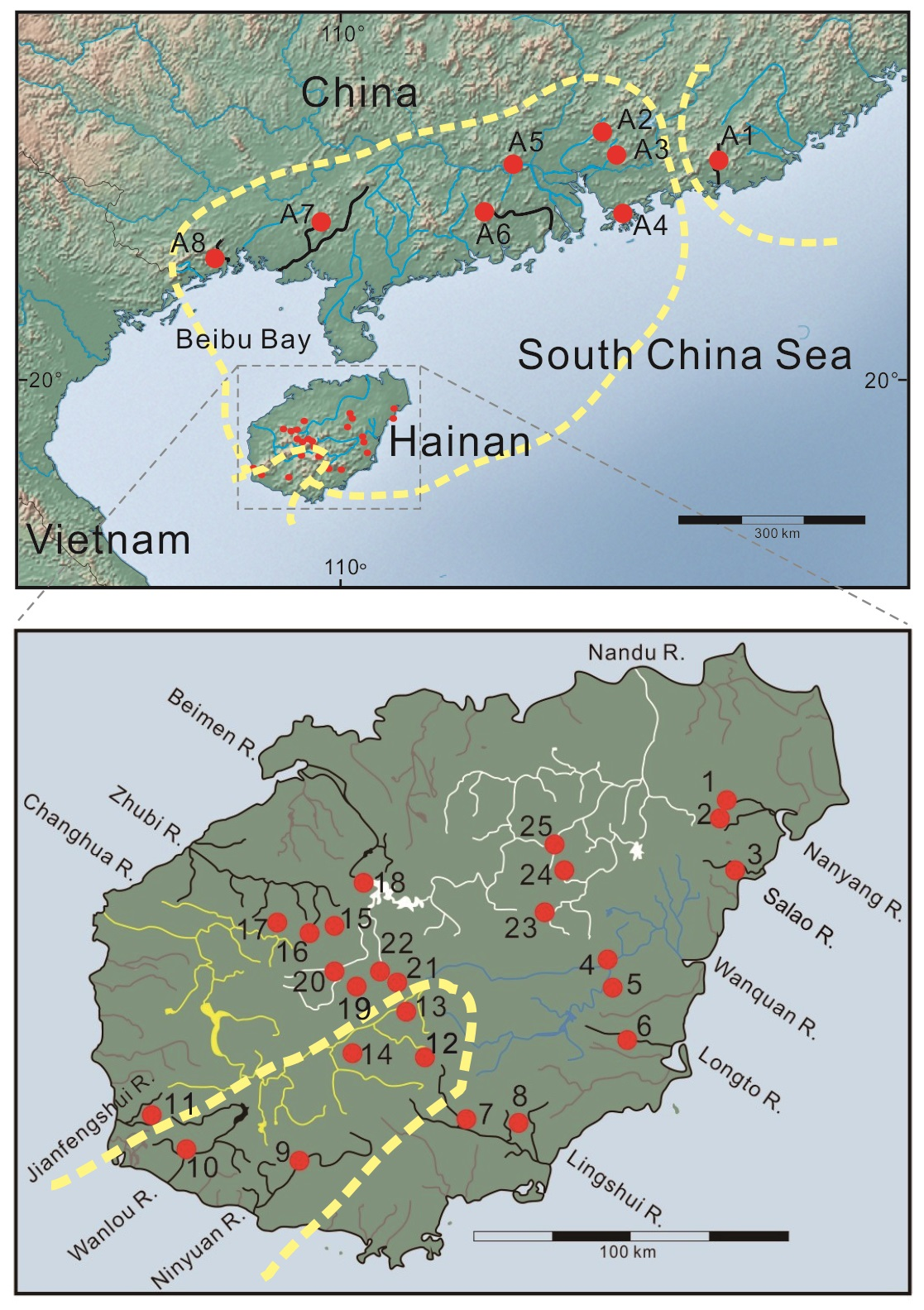

Supplement: S3 Fig — (TIF) [file pone.0282460.s004.tif]
