# Supplementary material for: Phylogeography of Aphyocypris normalis Nichols and Pope, 1927 at Hainan Island and adjacent areas based on mitochondrial DNA data
Source: PLoS One. 2023 Feb 28;18(2):e0282460. doi: 10.1371/journal.pone.0282460 (PMC9974131; doi:10.1371/journal.pone.0282460)
Supplement: Attachment — Submitted filename: Response to the reviewers.docx [file pone.0282460.s005.docx]

**In response to Reviewer’s comments**

Author: I-Shiung Chen and Nian-Hong Jang-Liaw

Manuscript Title: Phylogeography of Aphyocypris normalis Nichols and Pope, 1927 at Hainan Island and adjacent areas based on mitochondrial DNA data (PONE-D-20-37420R1)

We appreciate the reviewer’s comments and suggestions. The manuscript has been revised accordingly. The following are our answers to the comments:

**Major points:**

(1) Divergence time estimation:

L245–: Because the divergence time estimation is directly affected by the evolutionary rate, the validity of 1.0%/Myr should be explained, or some excuse is required for the resultant estimates. Also, in the Discussion part (L517–), more careful discussion is required for the divergence time.

Furthermore, I’m not sure whether the authors notice it or not, Huang et al. (2019) present about twice values for the corresponding estimates (e.g., tMRCA of A. normalis = 2.33 mya, 1.92–2.74). Huang et al. (2019) mentioned as “A molecular clock was calibrated using a divergence rate of 2% per million years (Yang et al. 2016)” and the basis of Yang et al. (2016) is “A molecular clock was calibrated using the divergence rate of 2% per million years, as previously postulated by Brown et al. [44] and Bermingham and Martin [45]”, i.e., very famous, classic papers’ values. Namely, Huang et al. (2019) should use the evolutionary rate of 1% (not 2% of the “pairwise” value, which should be half for one lineage), as same in this study. The discrepancy (twice values of the estimates) is quite strange. I simply guess that the Huang et al. (2019) misused the pairwise divergence value (2%/Myr) as the evolutionary rate directly, if the present study is correct. Similar mistakes can be found in not few papers. This can be mentioned to emphasize the value of the present revised analysis.

OUR REPLY—We had found that the difference of tMRC data between Huang et al.s’ result and ours. However, it was difficult to compare all the divergence time estimations in detail between these two works for they release very few information on it (only short description on divergence events order in the paragraph before “Discussion”). Yes, it seems a misusing as you mentioned. Your comment is valuable and we have added it in our discussion (L489-496). It is possible that the misusing is common in the results of other works like *Glyptothorax* (Chen et al. 2007), however, they did not provide clear, detailed information on their methods and results in the published papers as well. We cannot make comments on other papers on this issue.

(2) L344–, “suggesting that the differences in climates among biogeographic regions constitute effective isolation mechanisms to gene flow”:

This “suggestion” is not satisfactory. Correlation does not necessarily mean causality. Also, there are many differences between the boundaries in Fig. 3a and 3b.

Also in the Discussion part (L457–), I cannot understand the degree of congruence between climate division by He and Zhang [45] and phylogeographic division by this study, although I agree that geographic and climatic settings (topographical features, frequency of floods, etc.) have affected the population structure by disturbing the separation of basins that is an important basis of isolation. I believe that the latter claim has general value and should be emphasized in this paper. However, the evidence for the claim in this manuscript is still not sufficiently convincing.

OUR REPLY—In general, we will consider the vicariance of different rivers will be the main isolation mechanism for freshwater fauna. It does not work in Hainan; at least in the phylogeographic results of *A. normalis* in this study. We tried to find a possible hypothesis and finally found He and Zhang’ work (1985). We set and modified their climate division to identify our phylogeographic division hypothesis and test it by ANOVA. We compared the “Drainage by river systems” hypothesis (Scenario 3) and climate-modified biogeographic regions hypothesis (Scenario 4) in Hainan and found latter is a more reasonable scenario based on the AMOVA test (Table 3).

(3) “isolation by distance” is not well evidenced.

The authors contrast an isolation by distance scenario with a vicariance scenario (L501, L520–523, L531–). But they do not provide the evidence distinguishing these scenarios. Peripheral populations can be isolated also by “small” vicariance factors.

OUR REPLY—We identified the “by distance” and “by vicariance” of scenario following the possible connection between rivers (such as belong to large Pearl River System will be considered as “by distance”). As you mentioned, it is well accepted that peripheral populations can be isolated also by “small” vicariance factors. However, in a land/island suffered by serious flood events frequently, like Hainan Island or Pearl River system in southern China, the vicariance factors occurred by close-by rivers should be considered as a weak isolation mechanism in the long differentiation history of freshwater fishes there.

(4) The structure of the “Discussion” part is confusing.

Obviously, the paragraphs from L508–L536 should be included the preceding section (“Genetic diversity and structuring in Aphyocypris normalis”), since the paragraph is the discussion deeply relating to those just above. Only the paragraph from L537 may be separated (but see the below comment).

OUR REPLY—We had modified the title of this section to response your comment. Others please see our response to comment 5 below.

(5) Discussion of A. arcus (L537–546) should be shortened or removed. In the latter case, the results should be also removed from the main text and figures.

The phylogenetic relationships of Aphyocypris spp. have already shown in Huang et al. (2019) with the same type (mtDNA, but longer) of data. The only new knowledge is that the authors could not collect the A. arcus in Hainan Island. Nobody can conclude that A. arcus is not distributed in Hainan from this result of the sampling. A possible way to do it is to reexamine the specimens used in Du et al. [76] with comparative specimens and to show their morphological key is not effective to identify different species. In this paper, at least the expression of the suggestion should be weakened more.

OUR REPLY—Huang et al.s’ work (2019) did not touch the issue of population of *A. arcus* on Hainan Island. We (both of the authors and their team members) had excused more than 6 freshwater fish specimen collection trips in Hainan and over dozen collecting trips in southern mainland China. All the specimens used in this study were collected by our team members in the habitats, not purchasing from local markets (this is the reason why our sample size was small in some localities). For the corresponding author, he had a long trip in KuangXi Province for collecting freshwater fish specimen in 2002 with Pisces Division of Chinese Academy of Sciences, Beijing. He met several populations of *A. arcus* in Red River system in KuangXi and kept several specimens of *A. arcus* in NMNS, Taichung, Taiwan to public for later comparative studies like this work. We do not dare to say we are good enough to be experts on ichthyological fauna of Southern China, but we are sure that our collection efforts in Hainan were comprehensive enough to cover at least one possible *A. arcus* population. We found no any *A. arcus* in our specimen after the DNA check on all *Aphyocypris* species collected from Hainan. There will be arguments always that nobody can claimed that they had visit ALL possible *A. arcus* habitats in Hainan and have a conclusion on the absent of the species in the island based on their specimen collection results. However, we just provided our observation and comments on *A. arcus* in Hainan based on our data and experience. This will be a valid contribution on freshwater fish studies in this area we believe.

We have weakened our comment by adding a sentence in the end of the section following your suggestion.

(6) Discussion of A. moltrechti and A. amnis (L548–580) should be much shortened or removed, because no new information is given in this study for this matter.

I agree that any taxonomic problems should be resolved as soon as possible. However, the present data do not provide any new information to resolve this problem. This part is just the authors’ claim.

That is, (1) the low genetic diversity in the Zhoushui R. population and its small divergence from other populations have already been reported in Chiang et al. [77] using mtDNA and microsatellite data; (2) Liao et al. [62] provide the morphological diagnostic characters for A. amnis, which seem not to be distinct and hence controversial; (3) generally, taxonomic ranking (intraspecific variation, ESU, subspecies, or species) of allopatric populations with small morphological/genetic differences could be often subjective; and (4) there are biological species with no genetic differences from relatives in neutral markers. A possible way to conclude this problem is to publish a nomenclatural article to synonymize the A. amnis with integrating morphological and genetic data.

However, some taxonomists may consider that if a population can be distinguishable from others in any characteristics or combination of characteristics, it can be treated as a “species” and the species rank is more beneficial for conservation action than just a local population.

I do not dare to discuss this matter with the authors, but I only like to say that the part should be shortened or removed because no new information is given in this study and the discussion is not satisfactory to lead to resolution.

OUR REPLY—We agree with your totally. All the points you mentioned had been released at the same year that the new species *A. amnis* was reported. However, Liao et al. (2011) did not face up to the released information, and consciously ignored them. The identification key provided by Liao et al. (2011) could not effectively distinguish the new species from *A. moltrechti*, which caused difficulties for the follow-up fish researchers in identification. We agree that the promotion of a treated population into the species rank is more beneficial for conservation action than just a local population, but it should be based on a taxonomic truth, using the correct and nature taxa, then carry out its conservation strategy. From a point of view on DNA, the *A. amnis*/*A. moltrechti* populations from three rivers do show genetic differentiation between them (although very tiny comparing to other cyprinids like *A. normalis*). However, the need to promoted the taxonomic rank for the population in the Zhoushui R. to protected it should be re-considered carefully. The differentiation of freshwater fish in different water systems is very common, and it is impossible for us to divide all the populations that have mutations and rising their conservation level, especially those with such a small degree of variation. If taxonomies are not established based on the “true” speciation view, the precious funding, resources for conservation will be diluted and waste, and cannot be used on the species/populations that need to be protected emergency.

It is the responsibility of local ichthyologists to establish/correct ichthyological taxonomy in their land. We feel embarrassed that we have not been able to make a complete statement on this issue in a single paper. Herein we integrate some previously known but ignored information with few new data we provide to emphasis to this view and put it forward again. We do not expect to have a complete statement to reply to this issue, but we hope to remind Taiwanese ichthyologists that this issue is controversial, and we hope that there will be more complete reports on this issue in the future.

**Medium points:**

(7) L36, “The relationships of A. normalis between Hainan and mainland China populations are also discussed in this report.”

Again, I should say that the readers of Abstract want to know what is discussed.

OUR REPLY—We have modified the abstract following your comment.

(8) L161–: The section of “Data analysis” is better to be divided. The next section “Divergence times among major clades” is also “data analysis”; so they are unbalanced. The section of “Data analysis” includes two topics; i.e., “Phylogenetic analysis” (including two datasets) and “Population structure” (AMOVA and SAMOVA).

Also in the “Results” part, the results of AMOVA/SAMOVA (L321–377) should not put in “Phylogenetic analysis” section because they are not phylogenetic analysis. A separate section should be established.

OUR REPLY—We have modified this manuscript following your comments.

(9) L185–: In the explanation of AMOVA/SAMOVA, the authors should first explain all the models include two hierarchies of groups (i.e., groups of population samples and population samples). Some (schematic or realistic) figure for each model (even as supplementary) will be effective. Consider presenting it. It is not easy to understand the models only by text.

OUR REPLY—It is the major contribution of this work to describe the isolation scenarios revealed by the AMOVA/SAMOVA analyses. We have modified the manuscript to clarify the scenario reference and showed the result of SAMOVA with a new figure S2 following your comments (13) and (14) respectively. Your excellent comments help us to improve this manuscript a lot.

(10) around L246: The tree prior should be explained here. According to Table 5, the constant population model seems to be used. I strongly recommend that the analysis with other models, especially the Birth–Death model (see Ritchie et al. 2017: Syst Biol, DOI:10.1093/sysbio/syw095), should be also added and mentioned for showing the robustness of the results. This is because coalescent processes for the geographical samples of animals with low dispersal ability (like freshwater fish) should differ from those in a constant population.

OUR REPLY—In the first beginning of this study, we found the isolation mechanisms usual to freshwater animals (isolated by strait, separated between rivers, etc.) were not suitable in this study for we detected the closer relationships among populations between mainland China and Hainan Island, as well as between river systems. We decided to use a simple, neutral model to detect the MRCA in *A. normalis* in this study and finally we choose the constant population model. We have modified this section under your suggestion.

(11) L247, “for each partition”: There is no explanation on the partition of sequence data. Should be explained.

OUR REPLY—The “partition” is the DNA sequence data. We have modified this sentence to clarify it.

(12) L295, “NJ and ML trees (S1 Figure)”: The information of the branch length of the ML tree should be kept and shown. Also, bootstrap values should be shown also in S1 Figure.

OUR REPLY—We have modified the S1 Figure following your suggestion.

(13) L321–L345: Why does not the authors refer to the number of hypotheses here? What are the “two hypotheses” (L336) and “the last hypothesis” (L339)? The explanation here is difficult to understand. L321–323: This should not be a main topic (i.e., relation to hypotheses is unclear). Gene flow (L323) between which? It will much better to start to explain important issues.

OUR REPLY—We have modified the Table 3 to refer the “Hypothesis scenarios” in this study to response your comment.

(14) L359–: The result of SAMOVA should be shown as a figure (even if in supplementary).

OUR REPLY—We have made a figure (Figure S2) to response your comment.

(15) L396–: The term “Clade” is not suitable if it is defined in the haplotype network, because some are not monophyletic. The “Clade” should be those defined in Figure 2, shouldn’t it? Explain it first. Relating to this, not only Clade II but also Clade I is NOT monophyletic (L404).

OUR REPLY—We have modified the Network descriptions following your comment.

(16) L483, “Subclade Ia is currently limited mainly to the west-central mountain area and shows clear differentiation between nearby populations in ranges NW and SW. “

I cannot understand this part. The Subclade Ia is distributed in NW. Probably it’s Subclade Ib in C, according to Fig. 3b, isn’t it?

OUR REPLY—Yes it should be Subclade Ib in C. We have corrected the mistake.

(17) L496–, L519–: No figure is provided for haplotype (clade) distribution in the continental area (only explained in text). I wonder if such a figure also should be put in Fig. 3(c?).

OUR REPLY—We have made a figure (Figure 3c) to response your comment.

**Minor/Trivial:**

(18) General: A blank line should be inserted between paragraphs and between figure legend/table footnote and text, if no indent is used. It is difficult for reviewers to identify the paragraphs and to distinguish the text from explanation of figures/tables in this manuscript.

OUR REPLY—We have marked all legends of tables and figures in blue color to response your comment.

(19) General: The name of a river is usually accompanied by “the” (definite article). There are inconsistencies in many places.

OUR REPLY—We have modified the manuscript following your comment.

(20) L91, “with much lower sampling density”: Compared what? That does not yet appear. --> e.g., “with too low sampling density to clarify....”

OUR REPLY—We have modified the manuscript following your comment.

(21) L96: “within” --> “with”?

OUR REPLY—We have corrected it following your comment.

(22) L98: “a freshwater fish” --> “this freshwater fish”, “this species”.... Should be restricted.

OUR REPLY—We have modified the manuscript following your comment.

(23) L102: “proposed” --> “propose”

OUR REPLY—We have corrected it following your comment.

(24) L106: “were” --> “are”

OUR REPLY—We have corrected it following your comment.

(25) L109: “Sample used” --> “Samples used”

OUR REPLY—We have corrected it following your comment.

(26) L175, “15,000,000 trees”: This should be “15,000 trees”. (i.e., 2,000,000/100*0.75=15,000)

OUR REPLY—We have corrected it following your comment.

(27) L177–, “The genetic distances ... were calculated using MEGA5 with the Kimura 2-parameter model (K2P)”: In the response letter, the authors explaine the reason why the best model was not used as it (HKY+I) cannot be available in MEGA. There should not be large differences between the K2P and HKY because they are similar models (ti, tv, 2-parameter model). However, I wonder why the authors do not use other software that can use HKY+I to calculate the distances (e.g., PAUP*).

OUR REPLY—It is a pity that PAUP had no any update since 2000 though it was very popular for many phylogenetic researchers. More and more studies turn to use other programs such as MrBayes and MEGA. In my opinion, MEGA is a “growing” software and the last update (MEGA X) was released in 2018. It is popular to researchers currently for several basic sequence statistics available in MEGA, and the most of all, easy to use with friendly operational Interface. I (the corresponding author) decided to use MEGA in this work and said goodbye to PAUP after this work.

(28) L181, “which was carried out using ANeCA v1.2”: Should be deleted. TCS, which was actually used in this study, is an independent program. If ANeCA can automatically nest the network, the explanation should be changed. However, the result of the nesting is only partly shown in the Results (Fig. 5). Readers cannot see full results of the nested clades.

OUR REPLY—We had removed these words following your suggestion.

(29) L185, “The relative contribution...”: Change the paragraph. AMOVA/SAMOVA are quite different approaches from tree analyses.

OUR REPLY—We have modified the manuscript following your comment.

(30) L193, “as (hypothesis 3) and (hypothesis 4)”: Should remove parentheses.

OUR REPLY—We have modified the manuscript following your comment.

(31) L205: “suggests” --> “suggest”

OUR REPLY—We have corrected it following your comment.

(32) L206: “the hypothesis” --> “the relevant hypothesis”

OUR REPLY—We have modified the manuscript following your comment.

(33) L206, “SAMOVA 2.0 [48] to confirm that the grouped populations are geographically homogeneous and maximally differentiated from each other or not,...”: This explanation should be insufficient (inadequate) and reconsidered.

OUR REPLY—We have modified this sentence following your suggestion.

(34) L211, “We set the population of a single river system as a grouping unit (17 counts),”: What is the “population”? “population samples”?

OUR REPLY—Here the word “population” is similar to “sampling localities”. We add the latter one to clarify what we want to say.

(35) L213: “exam” --> “examine”?

OUR REPLY—We have corrected it following your comment.

(36) L214–, “However,... pattern).”: I think this sentence should be removed. Why “However”?

OUR REPLY—We have removed this sentence following your suggestion.

(37) L216–222: Redundant. This part can be shortened.

OUR REPLY—We have modified this section following your comment.

(38) L228: “are” --> “is”

OUR REPLY—We have corrected it following your comment.

(39) L269–, “Estimates of genetic variability differed accordingly.”: Not enough words, and not necessary. Should be removed

OUR REPLY—We have removed the incomplete sentence following your suggestion.

(40) L270–274, “Within-population ....(Table 1).”: Put this general description above the specific description (--> L264).

OUR REPLY—We have re-arranged this section following your suggestion.

(41) L286: The section title “Phylogenetic analysis” seems not suitable in the Results. For example, “Phylogenetic relationships” seems better.

OUR REPLY—We have modified the section title following your suggestion.

(42) L290: “including” --> “including those in”

OUR REPLY—We have modified this sentence following your comment.

(43) L318, “Distances”: Which distances? Should be clearly shown.

OUR REPLY—We have modified this sentence following your comment.

(44) L383–: “Like A. normalis and A. moltrechti, the two Aphyocypris species also formed...” --> “Aphyocypris kikuchii and A chinensis also formed...”

OUR REPLY—We have modified this sentence following your comment.

(45) L392, “Haplotype Network and variations among clades”: “Network” --> “network”. “variations among clades” --> What variation?

OUR REPLY—We have simplified the section title as “Haplotype network”.

(46) L394, “within haplotypes”: Impossible.

OUR REPLY—We have changed this word with “between”.

(47) L396, “MP tree”: No such analysis in this paper.

OUR REPLY—It was a misuse of “ML tree”. We have corrected it in the manuscript.

(48) L399, “a fourth”: “one of four sampling locations”? But there are eight localities on the mainland. Ununderstandable.

OUR REPLY—It meant that there are 4 locations which host the haplotype h4. Three of the locations are in Hainan (sites 4, 5, 23), and the last (fourth) one is in mainland (site A5). It shows that the distribution of h4 is over the strait.

(49) L400, “However”: Ununderstandable.

OUR REPLY—We have replaced the “However” by “Furthermore” in the sentence.

(50) L402: “, and included” --> “, which included” (h1 cannot include five haplotypes.)

OUR REPLY—We have modified this sentence following your comment.

(51) L405, L406, “the two haplotypes”, “these two haplotypes”: Clearly show them.

OUR REPLY—We have modified these sentences to response your suggestions.

(52) L414, “Numbers inside circles are haplotypes.”: Should not be shown in bold.

OUR REPLY—We have modified these words following your comment.

(53) L426: “Pleistocene” --> “the Pleistocene”

OUR REPLY—We have corrected it following your comment.

(54) L428: “tMRCA” and “tmarca” should be consistent.

OUR REPLY—We have corrected them following your comment.

(55) L439, “These two groups”: There are three groups (clades). What are the groups here?

OUR REPLY—We have modified this section following your comment.

(56) L455: “resulting” --> “resulted”?

OUR REPLY—We have corrected it following your comment.

(57) L476, “highly supported phylogenetic subclade”: Show the subclade in parentheses.

OUR REPLY—We have modified this section following your comment.

(58) L484: “Upstream Changhua River (range SW) populations show...” --> “Sharing Clade III haplotypes, the upstream Changhua River (range SW) populations show...”. (to add an explanation of the “close relationship”)

OUR REPLY—We have modified this section to explain why they shared close relationship.

--- end of comments
