# Supplementary material for: Phylogeography of Aphyocypris normalis Nichols and Pope, 1927 at Hainan Island and adjacent areas based on mitochondrial DNA data
Source: PLoS One. 2023 Feb 28;18(2):e0282460. doi: 10.1371/journal.pone.0282460 (PMC9974131; doi:10.1371/journal.pone.0282460)
Supplement: Attachment — Submitted filename: Response to the reviewers R3-OK.docx [file pone.0282460.s006.docx]

**In response to Reviewer’s comments**

Author: I-Shiung Chen and Nian-Hong Jang-Liaw

Manuscript Title: Phylogeography of Aphyocypris normalis Nichols and Pope, 1927 at Hainan Island and adjacent areas based on mitochondrial DNA data (PONE-D-20-37420R2)

Again, we deeply appreciate the reviewer’s great comments and suggestions on this work. The manuscript has been major revised accordingly as possible we can, and it had been modified completely almost. We accept almost all suggestions the reviewers offered, and some we need more time to reconsider them.

The following are our answers to the comments which we did not follow completely. Others were all accept by the authors.

**Major points:**

OUR REPLY—We have modified the manuscript following all your major comments.

**Medium points:**

(10) L184, Issue of genetic distances and “PAUP*”:

I believe that the evolutionary distance is better to be calculated under the best evolutionary model that is shown in the same paper. However, I do not dare to claim this again because the authors have already denied it twice.

Now the only thing I like to say is that the author’s explanation does not make sense. The authors indeed used PAUP* in this paper. Also, PAUP* has been continually updated until now (see https://paup.phylosolutions.com/), although the authors write that the software has stopped updating in the reply letter.

Again, I do not dare to request the change because the models will not largely change the results (distances) and the usage of K2P is not incorrect (only it is not the best). However, only the thing I want to say is the authors’ explanation did not convince me.

OUR REPLY—I understand your point that the K2P is not the best choice for distance analysis. As you see, we had applied PAUP to construct NJ and ML trees for this study. However, it was almost eight years ago that we started to prepare this manuscript. After that time, we had no update on this software for the compatibility with PC system was not so good then. We will consider to re-set up this software in our PC and try to work on the distance analyses in next revised version if you insist.

(11) L247, L462, Issue of the tree prior:

In the previous comments, I recommend that the authors should try to use also the “Birth-Death” model as a tree prior (see Ritchie et al. 2017: Syst Biol, DOI:10.1093/sysbio/syw095) because the constant population size model is the coalescent model for a practically single population. The use of the latter simple model seems to be inconsistent with the population differentiation claimed in this paper. The authors explain something in the reply letter, which did not convince me. Because this is the authors’ paper, I do not dare to repeatedly comment on the same thing. However, I only want to say that the use of the constant population model alone may not be the best choice.

(12) L256, “The parameters of the substitution model GTR were estimated independently for each haplotype data.”: Although the authors note that they estimate the model parameters independently for each haplotype, I have completely no idea what they mean by that. Since this study uses only one gene (cytb), there must be only one set of parameters for GTR. Or, since it is a protein-coding region, it is possible to give three different models for each codon site. The authors should provide a clear explanation.

OUR REPLY—For the comments 11 and 12, we need more time to discuss the detailed of such technical suggestion. Please allowed us to reply formally in the next revised version.

(13) L320–330, Figure S1: The branch length should be reflected in the ML tree. Why did the authors remove the length information only from the ML tree? Strange.?

OUR REPLY—We had scaled the length of the ML tree in Fig. S1. Please recheck it again.

(15) L391–396: This part is a discussion, which should be moved to the “Discussion” part. Also, readers are surprised when Indochina is suddenly mentioned. It should be properly explained in the “Introduction” and the “Materials and methods” parts.

OUR REPLY—We moved L391-396 to “Discussion” part (L588-593 in the revised manuscript) following your suggestion. In the “Introduction” we introduced the distribution of A. normalis in Vietnam in L82, 88-90 in the revised manuscript.

(17) L605–, “since only four haplotypes were identified from 22 sequences that were 1,091 bp long and only one haplotype was found in the Dajia and Wu Rivers’ population”:

The number of haplotypes neither supports nor denies a different species. Should be rewritten.

OUR REPLY—We deleted the words “However, our data do not support the specimens collected from the Zhoushui River being a different species, however, since” to make the paragraph more readable.

(18) L627–629, “We suggest that both....in the future.”: What’s happened? Is this “suggestion” for A. amnis issue? How related? Should be rewritten.

OUR REPLY—In the manuscript we ended it with the *A. amnis* issue. We modified this sentence as “We suggest that more appropriate nuclear markers should be used to evaluate the validity of *A. amnis* in the future.”

**Minor/Trivial points:**

(31) L102: “comprehensive sampling efforts across the species’ entire range” --> “comprehensive geographic sampling”. Redundant and not very precise (how about Vietnam?)

OUR REPLY—We had modified this sentence to make it more readable. (L101-104)

(45) L321, and Figure 2, “>90%”: As the authors may know that the Bayesian posterior probability is usually expressed as 0.00–1.00 as probability, not in %, although I do not dare to request to change it.

OUR REPLY—We had modified the Fig. 2 following your suggestion.

(46) L325–, “Abbreviations beside clades or sub-clades correspond to the sampling sites listed in Table 1.”: I cannot find such abbreviations.

OUR REPLY—They are I, II, III and Ia, Ib, Ic, and the description should be “Abbreviations beside clades or sub-clades correspond to the **clades** listed in Table 1.”

(47) L338, “the two a priori phylogenetic hypotheses that we examined”: There are four hypotheses. Also, the part “, isolated by the strait (Scenario 1 in Table 3) and drainage by river system (Scenario 2 in Table 3),” seems not clear. Please rephrase this sentence.

(48) L339–363: Replace “scenario” by “hypothesis”, as is in “Methods”. Confused.

(50) L341–343, “we found in both hypothesis... of the Hainan Island vs. mainland hypotheses”: Contradicted. The last part should be for only Hypothesis 1, not both.

(52) L348–, “Our second set of experiments...”: In fact, the part below describes scenarios (hypotheses) 2 and 3. What’s happened?

OUR REPLY—These hypotheses were really confusing. Very sorry. We had re-written this section and re-named the hypotheses (1a, 1b, 2a, 2b) in L340-346. Please give us your suggestion on it.

(54) L354, “low”: Are the 0.32773 and 0.51140 really low? Strange.

OUR REPLY—It was compared to another hypothesis (2b). We had modified the sentence. (L439)

(55) L358, “(Scenario 4 in Figure 3)”: In Figure 3, no word of “Scenario 4” appears. Strange citation.

OUR REPLY—It should be “Table 3” and we had corrected it.

(59) L378, “ FST values correspond to among individuals within populations.”: This is quite wrong.

FST in AMOVA is the variation component assigned to populations (without group division). So, 1–FST is the value that corresponds to the variation component among individuals within populations, isn’t it?

OUR REPLY—We had modified this sentence to make it more readable. (L359-361)

(68) L422, “A haplotype network is helpful in understanding the structure of phylogenetic relationships between haplotypes of Aphyocypris normalis among populations”: Should be rephrased. Strange. What is “the structure of phylogenetic relationships”? Phylogenetic relationships of what? “Between haplotypes”, or “among populations”?

OUR REPLY—We had simplified this sentence to make it more readable. (L373-374)

(69) L424, “two groups were erected”: What are these? Specify. First, these two groups should be defined (explained) based on the network.

OUR REPLY—We separate these two group with red broken line in Fin. 3.

(105) L594–, “More comprehensive comparative studies of A. arcus on Hainan Island both on DNA and morphology are necessary in the future.”: Strange. In the sentence immediately above, the authors state the possible absence of this species in Hainan, so a comprehensive comparative study may not be possible.

OUR REPLY—Agree. We removed the words “on Hainan Island”.

--- end of comments
