# Supplementary material for: Phylogeography of Aphyocypris normalis Nichols and Pope, 1927 at Hainan Island and adjacent areas based on mitochondrial DNA data
Source: PLoS One. 2023 Feb 28;18(2):e0282460. doi: 10.1371/journal.pone.0282460 (PMC9974131; doi:10.1371/journal.pone.0282460)
Supplement: Attachment — Submitted filename: Response to the reviewers R4-OK.docx [file pone.0282460.s007.docx]

**In response to Reviewer’s comments**

Author: I-Shiung Chen and Nian-Hong Jang-Liaw

Manuscript Title: Phylogeography of Aphyocypris normalis Nichols and Pope, 1927 at Hainan Island and adjacent areas based on mitochondrial DNA data (PONE-D-20-37420R3)

The following are our answers to the comments from reviewers.

**Reviewer #2:**

L108-L111, The title of manuscript is “Phylogeography of Aphyocypris normalis Nichols and Pope, 1927 at Hainan Island and adjacent areas based on mitochondrial DNA data”. The author mentions the phylogeographic structure of A. normalis in Hainan Island and mainland China. However, the authors gave an interesting conclusion as“We suggest that more appropriate nuclear markers should be used to evaluate the validity of A. amnis in the future（L644-L646）”. As the specimen of Aphyocypris normalis Nichols and Pope, 1927 were from Hainan Province, and “A. amnis” were sampled in Taiwan Province, what is the evidences? And is this relevant to the topic of this MS.

OUR REPLY— In this work we selected A. moltrechti, a species close to A. normalis both in morphology and ecology, as an outgroup to discuss the phylogeny of A. normalis. There might be another better choice rather than A. moltrechti occurred somewhere around Hainan Island, however, the most suitable samples we have in hand was the A. moltrechti. The A. amnis is a sibling species of the A. moltrechti and we included it in the outgroup as well. We found that there was no obviously difference between A. moltrechti and A. amnis based on the DNA data of this work. We mentioned this opinion in the last end of this manuscript, to point out that more researches are necessary in the taxonomy of this group, even in the Eastern Asian ichthyological fauna. This is not a main goal of this research; however, it is a valuable issue to discuss for local ichthyology society, which the authors of this manuscript belong to. We are not dare to overthrow the validity of A. amnis in such short comments. We described what we found in this work.

L129, Why does the author combine populations into 17 groups as AMOVA analysis?

OUR REPLY— We combined populations into 17 groups based on drainage basins or close geographic proximity (L226-229).

L236-L238 “we combined populations into 17 groups based on drainage basins or close geographic proximity (i.e., the following populations were combined: sites 1–2, 4–5, 7–8, 12–14, 15–17, 19–25, and A2–A5; see Fig. 1).”

L285, “15 (1.283%) on the Zhubi River, which was represented by only two specimens”, why not use all the 7 specimens in the Zhubi River, and the author use 7 individuals in the AMOVA analysis.

OUR REPLY— These are two different scales for discussion. One is by “population (AMOVA)”, the other is by “site location (Hd)”

L348-L349, Table 2, Clade Ⅰ is an unresolved tree. Clade Ⅰa, Ⅰb and Ⅰc are unresolved trees and inappropriately defined as monophyletic group. This is quite doubtful.

OUR REPLY— We agreed with you that Clade Ia, Ib and Ic are not resolved well and inappropriately defined as monophyletic groups. So, we assigned them as sub-groups. The Clade I is supported by Bayesian and NJ analyses as a monophyletic group.

L418-L423, The author shows the phylogenetic relationships between A. normalis and closely related cyprinid species. But the author does not mention the phylogenetic relationships in discussion.

OUR REPLY—This section provided a rough overview of A. normalis and its siblings. Its most phylogeny close species is A. moltrechti, which is distributed in Taiwan Island, and we chose it as outgroup.

L485-L488, The reference (71) mention the phylogeography of Rana sauteri (Ranidae). R. sauteri is an amphibian, not freshwater fish.

OUR REPLY— The reference provides a case that “different climate and rainfall causing diverse breeding strategies”, no matter what animal it is. We cited several works focusing on animals other than fish in this manuscript, such as earthworms [20], a freshwater crab [21], freshwater fishes [22-29], a babbler [30], the peacock pheasant [31], deer [32], and ferns [33, 34].

L488-L491, The freshwater fish and the amphibian are the same in ecology?

OUR REPLY— The larval stage of amphibians is limited in freshwater habitats and isolated by lands between rivers, just like the freshwater fish. Yes, they can move on lands in their adult stage, anyway, their phylogeographic patterns are affected by freshwater habitats deeply. The freshwater fish and the amphibian are not the same in ecology totally but they are the same for being a good model species to discuss that how the river systems shape the fauna.

L496-L512 According the phylogeny and network, the results do not support the eight phylogeographic ranges of A. normalis. The author must present enough evidence to prove it and explain in detail.

OUR REPLY— In this discussion, we did add subjective opinions to the discussion of zoogeographic zoning of this fish based on the climate zoning of Hainan proposed by He and Zhang [66]. This model is more reasonable than the well-accept one “shaping by the isolation of rivers” in this case. We had discussed this opinion in this manuscript.

L517-L519, The author must explain why the same river system can be divided into two groups. What is the reason for the distinction?

OUR REPLY— Some river systems in Hainan are large and complex. The isolation mechanism of freshwater animals is not clear and well understood. For example, the main stream of river systems itself could be a barrier for some small freshwater fish species which hide in small tributaries. And, climate might be a force to shape the characters of genetic structures of freshwater animals. In the latter case we provide a case on an anura Rana sauteri [75] for refernce.

L527, Subclade Ib is unresolved tree.

OUR REPLY— The grouping of this clade is supported by BI/NJ/ML. We considered this grouping in subclade level.

L534-L542, There are many studies of freshwater fish focus on the phylogeographic role of Changhua River. Needs to added the latest refs.

OUR REPLY— We cited some latest works focus on phylogeography of Hainan freshwater fish in the revised manuscript following your suggestion.

L571, Subclade Ia is unresolved tree and not a distinct monophyletic Clade. How about the h46?

OUR REPLY— Subclade Ia is supported by Bayesian analysis as a monophyletic clade among Clade I. H46 is outside from Subclade Ia.

L572-L573, Subclade Ic is a distinct monophyletic Clade. How about the h1 and h3? Why didn't the author combine the h1 and h3 in Subclade Ic?

OUR REPLY— The supporting of grouping between h1, h3 and other Subclade Ic (by all three analyses) is relative weaker than that among Subclade Ic (only by Bayesian analysis). Besides, all the Subclade Ic are distributed in Dong R. (Pearl R.), mainland China; the h1 and h3 are in Nanyang R., Hainan Is. The grouping of Ic are supported both by DNA data and their distribution.

L616-L646, Why does the author discuss the other A. species in Taiwan province? not concerning to this MS.

OUR REPLY— Please refer to our answer reply to the first question. (L108-L111, The title of manuscript……)

**Reviewer #3:**

The reviewer was asking to use up-to-date softwares, or models. As I see, the authors are willing to use updated software, still they did not use it. The journal is PlosOne having high standards. So, if I were the lead author of this manuscript I would immediately recompute the results using better model even if the data is 8 years old. I would also try the suggested models to see what is the difference of the outcomes of the reported and the suggested models.

Additionally, rewriting of the sentences sometimes remained as confusing as previously were. I suggest to consult with more colleagues, go through the entire manuscript. Involve new eyes into the quest for such sentences.

OUR REPLY—In this version we had asked Dr. Graham L. Banes, associate scientist in the Wisconsin National Primate Research Center at the University of Wisconsin–Madison for proof-reading the revised manuscript. We sincerely thank you for your expectation and assistance in this work.

--- end of comments
