# Supplementary material for: Phylogeography of Aphyocypris normalis Nichols and Pope, 1927 at Hainan Island and adjacent areas based on mitochondrial DNA data
Source: PLoS One. 2023 Feb 28;18(2):e0282460. doi: 10.1371/journal.pone.0282460 (PMC9974131; doi:10.1371/journal.pone.0282460)
Supplement: Attachment — Submitted filename: Reviewers reviews R5.docx [file pone.0282460.s008.docx]

**In response to Reviewer’s comments**

Author: I-Shiung Chen and Nian-Hong Jang-Liaw

Manuscript Title: Phylogeography of Aphyocypris normalis Nichols and Pope, 1927 at Hainan Island and adjacent areas based on mitochondrial DNA data (PONE-D-20-37420R4)

We deeply appreciate the reviewer’s great comments and suggestions on this work. The manuscript has been major revised accordingly as possible we can, and it had been modified completely almost. We accept almost all suggestions the reviewers offered. The following are our answers to the comments.

**Reviewer #2:**

>The authors made an interesting topic in this MS, and I especailly apprecaite their detailed samling work and analysis on Wuzhi & Yinggeling, beyond the formers' study. However, the authors did not give more discussion on their differences and possible explanation. Here is another work focus on the same topic, pleased read it and discuss. Wei et al. 2021. Population genetic structure and phylogeography of Aphyocypris normalis on Hainan island and its neighboring regions. Chin J Appl Environ Biol, 2021, 27 (1): 191-199.DOI: 10.19675/j.cnki.1006-687x.2020.01025

**OUR REPLY**—Thank you for your useful information. We update our manuscript with the recent reference you provided (it should be Li et al. 2021). We reconsidered the possible isolation mechanisms occurred by the Yinggeling and Wuzhishan Mountains in accord with Li et al. s’ (2021) work in LN 476 -481.

>The authors gave interesting workd by proposing hypothesis in explaining the phylogenetic pattern of A. normalis. As the resecrch focus in this MS,the details of the hypothesis as well as why giving such a hypothese are need, e.g., in hypothesis 2, the ciatations on why draniage or climate could be used as the controlled factor; the chracters of 8 climatic zone and its relation to freshwater fauna; what are the 12 drainages and their geological developmental traits; either in introduction or in discussion.

**OUR REPLY**—We had modified the manuscript following your suggestion. We introduced the climate division in Introduction by adding “Moreover, we introduced a climate division hypothesis [36] to discuss the possible isolation mechanism on freshwater fauna in Hainan Island” (LN103-104) in introduction. In LN54-60, we had a short introduction on Hainan’ s rivers already. We also marked the characters of the climate divisions following He and Zhang [36] in LN 492-498 following your suggestion.

>Line 487. what does the logic of this sentence "Nonetheless, .. fit...". Thus, did the results by Cytb fit/unfit the division of climate? This was a really problem need the authors to clarify seriously.

**OUR REPLY**—Our cytb data fit their division of climate with high similarity. We had removed the “Nonetheless,” from the sentence.

>The result of 8 pholographic ranges was one of the two importants conclusion of this MS, seen from Abstract. Unluckily, I am puzzled by the authors work and explanation.

First, the details of samling sites needed illustration: climatic zone, river, corresponding to hypothesis 2b as sites (Just seen from figure 1 and figure 5a, I am not sure whether sample sites cover all the 8 climatic zones). Maybe still one more info, why the significant differences in site number in different climatic zones (only 1 site in a climate zone but many in another).

**OUR REPLY**—Please see the Figure 5. In 5a, the original climate division proposed by He and Zhang (1985) was presented; In 5b, we marked our sampling sites on a modified phylogeography-climate map. The sampling sites were uneven on these modified phylogeography-climate zones, but not “significant differences” as you mentioned; the numbers of sampling sites are 1 (1 zone), 2 (1 zone), 3 (3 zones), 4 (2 zones) and 5 (1 zone). We collected all the used specimens by ourselves and we visit all the habitats (sampling sites) displayed in this manuscript. In general, it is reasonable to be “uneven on sampling sites” for a work used materials collected from wild, for we cannot control the nature distribution pattern of the species we’re working on. We can say we did our best on sampling in the phylogeographic research of this fish.

>Second, seen from the figure 5a,b, the climate zone in Hainan Island was not concistent with the result of eight phylogeographic divisions. For example, climate 1 was divided into E and SE, climate zone 6 covered W and west part of SW; that means,the climatic division and the phylographic divisions intersected. As the authors suggested, the results in eight pholograhic ranges were moderated from climatic zone, so how to mederated, and the criteria? For example, the authors cancaelled the Subcalde (but in subgroup) in Clade I, then would it be suitabale to used subgroup to determine phylographical division?
The anthors took the results granted, too far-fetched without a equal criteria,and seemed eliberated to give such a result only aiming to match the 8 climatic zones for a possible explanation.

**OUR REPLY**—We agree with you point. Yes, in this work we proposed an incomplete, premature hypothesis based on the result of only one species. We do need more evidence to solid the phylogeography-climate zones hypothesis of Hainan island. However, it is difficult to be “complete” for a novel idea being proposed in the first beginning. In this case, we do not have any references to support our phylographical-climate division. This work is the first one to link ecological climate information and DNA phylogeny data in the species on Hainan Island. We are looking for more discussion, both agree and dis-agree ones, on this issue in the future.

>Third, the as an important conclusion, the author needed to give direct and clear result on the eight ranges by cytb, possibly directly marked in figure 2 or figure 3.
Moreover, adjacent areas were also inportant in this paper (the word also in the title), so the spatial pattern of A. normalis (clade II) also needed clarified and explanation, a 9th range or more? also controlled by climate or other factors? Should the factors controlling the phylographic range of A. nomalis be same or different in Hainan Island or adjacent areas?

**OUR REPLY**—The readers can connect the result on the eight ranges and cytb data by the linkage of clade/subclade identification. Please check the Fig 2 for the phylogeny position of each clade/subclade and check the Fig 5b for the location of each clade/subclade in the phylographical-climate ranges. Beside, we made a new Figure S1 to show the four division hypotheses.

For the phylographic range of *A. nomalis* in adjacent areas out of Hainan Island, it is a huge issue. Our data/sampling effort is far from “enough” to have a clear view on the phylogeography of A. nomalis in this area. For example, the Pearl river system is very complex with long ranges and abundant tributaries, and we only have 24 samples collected from 4 sites in this river system. It could be (and should be) a complete research topic to discuss the phylogeography of *A. nomalis* or any other species in Pearl River system. According to our limited sampling effort in this area, we only can discuss the relationship between southern China and Hainan’s populations (a.k.a., the isolation mechanism of the Qiongzhou Strait), but detailed phylogeographic comparison of populations of each river system. If you insist, we can modify the title of this manuscript by removing the “and adjacent areas”.

>In clade I, according to answers to the comments, the authors cancelled the validity of subclade and used subgroup, please giving personal difination on subgroup; otherwise, it is still cause confusion, as "group is monophyletic".
Moreover, in discussion the author still kept Subclade(since Line 562). I do think the author need to revised the paper in a throughout instead of only anwere the reviewers' comments and just simple corrections.

**OUR REPLY**—We did not use “subgroup” in our manuscript.

>Why add a part of comment on A. moltrechti, and discussed a validity of a new species A. amins? It is non busisness of the thesis of this paper. It needs another complicated work in another paper, using works following by taxonomic standarss, including morphology, molecular,evolotion,as well as possible geological and ecological evidences, but not only in discussion.

**OUR REPLY**—This work reports the phylogeography of a cyprinid fish distributed mainly in the southern part of China and Hainan Island. As you mentioned, the comment on outgroup species *A. moltrechti* complex was not priority of this work. However, the argument of the validity of *A. amins* indeed exists and no researchers pay efforts on it before for the “new species” can be found only in a very small stream, which means it is difficult to write a good paper with limited sample size of this species. The argument becomes not a priority for the researchers who need SCI papers to keep their academic job (or you can say, to survive in the reality world... ).

We believe that it is the responsibility of local ichthyologists to establish/correct ichthyological taxonomy in their land. We feel embarrassed that we have not been able to make a complete statement on this issue in a single paper currently. Herein we integrate some previously known but ignored information [85] with few new data we provide in this work to emphasis to this view and put it forward again. We do not expect to have a complete statement to reply to this issue, but we hope to remind Taiwanese ichthyologists that this issue is controversial, and we hope that there will be more complete reports on this issue in the future.

Reviewer #3: The authors answered the fellow researchers' questions as well, they rewritten the manuscript.

I suggest the manuscript for publication.

**OUR REPLY**—Thank you very much!

5. Review Comments to the Author (minimum 200 characters)
This manuscript by IS Chen and NH Jang-Liaw is the fourth revised version of PONE-D-20-37420, which reports the mtDNA phylogeography of a cyprinid fish, Aphyocypris normalis, commonly distributed mainly in the southern part of China including Hainan Island, based on an extensive geographical sampling. I have read this manuscript four times (except for the last one before the current manuscript).
Frankly, I am surprised at the number of problems that still remain in the current manuscript (5th round!!) and especially at the lack of care taken by the authors in the structure of the paper. Since this is probably my last comment, I would very much hope that the author will properly understand the meaning of my comments and make adequate revisions for the acceptance of this laborious work. I look forward to reading this paper after it is properly revised, completed and published.

>(1) L218–: The explanation for AMOVA analysis (models compared) is difficult to understand and should be improved.
The major problem is that the division of the regions according to Hypothesis 2b is not indicated in the Methods nor in the Results, but appears only in the Discussion. It is understandable that Fig. 5a cannot be repeated in the text, so perhaps the most reasonable way to do would be to illustrate all four models (1a, 1b, 2a, 2b) as the Supplementary figure. This would not only improve the fact that the division under Hypothesis 2b is not clear to the reader until the end of the Results, but it would also allow the reader to visually understand the differences between the models, which is complicated by the words alone.

**OUR REPLY**—We made a new figure set (Figure S1) to explain the four hypothesis models (1a, 1b, 2a, 2b) following your suggestion.

>Also, the below minor problems should be improved:
(1-2) L223, “mentioned” --> “explained”

(1-3) L223–, “To evaluate movement...”: I cannot understand what is beginning to be explained here. I guess that the sentence explains the hypotheses 1a and 1b first. If yes, the sentence should be like “For the hypothesis 1a, all sampled populations were combined into two groups representing the localities of their origin to evaluate movement across the strait. Alternatively, to evaluate movement between river systems (1b), we...”
(1-4) L230, “Moreover...”: This is not understandable. It should be, e.g., “For the hypotheses 2a and 2b, we evaluated movement...”. Also, remove “also” in L232.
(2) L116: “across the entire geographic range of the species”: Conflict to the statement in L85–88 (i.e., not entire). For example, add “almost”?

**OUR REPLY**—We had modified the manuscript following your comments.

>(3) L198–201: “We assumed the constant population size setting as a coalescent tree prior, which is...”:
This setting should be too simple to apply to subdivided populations with complex history even in a species. If I were the author, I would compare the results under different models (e.g., Birth–Death model, as suggested by Ritchie et al. 2017: <https://doi.org/10.1093/sysbio/syw095>) and show the robustness of the results. However, I would like to leave this point to the authors’ decision. (Oh, I noticed that I have made the same comment at least three times... Every time I seem to find this problematic...)

**OUR REPLY**—In our opinion, the phylogeography research is based on a number of assumptions that can only be known by inference and not repeatable (eg; past geological chronological changes, the happened evolution procedures…). The analysis and statistics of this kind of research are also constructed in many hypotheses, and various model hypotheses are constantly being proposed. However, there may be only one true (nature) evolution history. We understand that the goals of all “survived” models are trying to revise their inferences to the closest version of reality/nature, and no single model can fit all assumptions. As you suggest, comparing different models to get the most likely hypothesis from them is the general recommended drafting thinking. But we don't think the purpose of this work is to discuss the modeling/methods, and such comparisons would make this manuscript be out of focus. In addition, in our experience, we found that the simplest model always can lead to a similar inference as other new models. Therefore, we tend to choose simpler models or parameters instead of new models companied with many parameters that we really do not understand completely. In other fields of research, such as microbiology, where the model is not the key to the discussion of their results; simple analysis as MEGA without modeling can be acceptable (eg, https://link.springer.com/content/pdf/10.1186%2Fs42483-021-00103-z ). The authors can focus the manuscripts on their findings rather than on differences in methodological models.

>(4) L253–259: Move to the “Materials and methods”. These are not results. Especially, L255–257 are just repetition of materials. The result can start from L259, e.g., as “All sequences obtained from 127 Aphyocypris normalis and 22 A. moltrechti specimens were 1,091 bp in length”

**OUR REPLY**—We had modified the manuscript following your suggestion.

>(5) L295, Figure S1: The branch length should be reflected in the ML tree. Why did the authors remove the length information only from the ML tree? Very strange. I may have made this point before, too.

**OUR REPLY**— The ML tree we provided before showed a better shape. However, we replaced the ML tree following your suggestion.

>Also, in the legend of Figure S1, the support values should be explained.

**OUR REPLY**—We had modified the legend of Figure S2 following your suggestion.

>(6) L327–333: What is this part at all? This is not the results of “Phylogenetic relationships” and just what is explained in the method. Completely unnecessary. Should be deleted.

**OUR REPLY**—We had modified the manuscript following your suggestion.

>(7) Table 3: The place is quite inadequate. This table of AMOVA should be put in the “Population structure” part (L401–), so the table number should be renumbered (it will become Table 4).
(8) L402–407: Why are these results of phylogenetic relationships placed in the “Population structure” part? Completely incomprehensible. Move to the last or adequate part of “Phylogenetic relationships”.

**OUR REPLY**—We had modified the manuscript following your comments.

>(9) L408–410: Confused. Why is the matter of Fst explained here? No meaning. It DID NOT “imply few gene flow between tested groups”. Fct is the statistic to be focused. However, for Fct, it is not significant in all models (1a).

**OUR REPLY**—We modified the sentences following your comment; and we had more statements on FCT in SAMOVA.

>(10) L418, “but also not be true”: Too strong expression. This kind of models is not completely true or wrong. Rephrase it.

**OUR REPLY**—We modified it to “but also might not be true.”

>(11) L423–432, “The FCT value of Hypothesis 2a....within groups (Table 3): This section is confusing and inappropriate for context and explanation.
For example, these 10 lines can be... “(Delete the 1st sentence) The results of Hypothesis 2a, as well as Hypothesis 1b, did not strongly support genetic isolation between river systems. The last hypothesis (2b), dividing the region into eight biogeographical areas based on climate revealed that most of the variation was between groups (70.15%; Table 3[-->4]), which indicates that the hypothetical biogeographic region division is a strong barrier to gene flow. (Delete the last sentence because it is just an unnecessary description shown in Table.)”

**OUR REPLY**—We had modified the manuscript following your comment.

>(12) L477–479 (such as the elevation of different habitats after climatic events....strategies): Sorry, but I don't understand this part at all.

**OUR REPLY**—In a former study of the corresponding author, the phylogeographic pattern of a frog *Rana sauteri* is possibly formed in a large part by reproduction isolation formed by difference of climate characters between habitats, not by geographic barriers only [76]. It reminded us to check possible mechanisms which formed the phylogeography of animals other than geographic barriers. In this study, we checked the climate pattern of Hainan Island and tried to introduce it to our discussion.

>(13) L532, “they are different from other populations”:
The authors seem confused between the groups and phylogenetic relationships of mitochondrial DNA haplotypes and the cohesion and relationships of populations. Although the results of mtDNA can show evidence of river capture, it is still unclear that the fish in this region compose a historical population group and it is different from other populations. At a minimum, the expression should be softened as, e.g., “They are possibly different from...”.
Furthermore, it should be clearly stated somewhere (e.g., here), “However, because our inferences were based on mtDNA only, nuclear genome data should be examined for further information on population relationships and history.", or like this.
This kind of confusion is found also in L579 (“a unique phylogenetic group (Clade III) composed of populations of...”)

**OUR REPLY**—Good suggestions. We had modified the manuscript following your comments.

>(14) L577–, “in A, normalis”: Too narrow. How about replacing by “even in freshwater fish”?
(15) L586–594: “Huang et al. [25]...”: It is better to remove this paragraph.
In my previous comment to indicate this “inconsistency”, I made a mistake at the end (Sorry!), and this manuscript seems to inherit that mistake. If Huang et al. used the 2%/Myr clock (twice faster clock), the estimated time should become short (not long!), shouldn’t it? So, although the explanation in the Huang et al. is quite unclear and the result is inconsistent with the present one, I think those should be left alone without mention.

**OUR REPLY**—We deleted the section following your suggestion.

Minor/Trivial points:

>(16) L21: Delete commas before and after “Aphyocypris normalis”. Here “the freshwater fish” is not equal to “Aphyocypris normalis” but used adjectivally.
(17) L36, “LGM”: Spell out (i.e., “Last Glacial Maximum”)
(18) L157, “MEGAX” --> “MEGA X” (insert a space)
(19) L183, “and is shown herein (see Fig. 4)”: Should be removed. Unnecessary.
(20) L236: Change paragraph before “In addition”. The topic is changed here from AMOVA to SANOVA.
(21) L238, “or not”: Should be removed. Unnecessary.
(22) Several sentences in the results are sporadically written in the present tense, but should be written in the past tense (e.g., L259, 288, 291, 360, 362, 363, 542...). Check throughout.

**OUR REPLY**—We had modified the manuscript following your comments.

>(23) L323, “haplotype” --> “sequence”

**OUR REPLY**—It should be “haplotype”. The number of “sequences” (all samples’ sequences) is different from “haplotype”. We used haplotype data in that section, not all samples’ sequences.

>(24) L323, “0.75...”: Make the text so that it is clear which is the value of which clade.
(25) L324, “inferred from haplotypes”: Remove. Unnecessary.
(26) L325, L423: Insert a space between words (figures).
(27) L340–, The second column for Hypothesis 2b: “Hypothesis” --> “Hypothetical”?
(28) L357–358, “A haplotype network is...relationships.”: Completely unnecessary. Should be removed.
(29) L370, “In our haplotype network”: Unnecessary and Should be deleted. “The haplotype h1 was...”
(30) L376, “five” --> “five or six”
(31) L379, “each clade” --> “haplotypes”. Not “network for each clade”.
(32) L390–391: Indicate which values are for which clades.
(33) L411–, “hypothesis” (Methods) or “Hypothesis” (Results)? Either one should be used.
(34) L474, “concludes” --> “conclude”
(35) L475, “by the geographical” --> “solely by the geographical”: It is wrong to deny it completely.
(36) L487, “Nonetheless, their divisions” --> “Their divisions”. The context is not connected at all with the word "Nonetheless."
(37) L579, “Moreover”: Delete. The context is not connected at all with the word "Moreover" in the changed paragraph.
(38) L582, Cite a reference [19].

**OUR REPLY**—We had modified the manuscript following your suggestions.

>(39) L586, “MRCAs” --> “tMRCAs”
(40) L592, “may” --> “should”

**OUR REPLY**—The section had been removed following your another suggestion.

>(41) L598, “between A. normalis and A. arcus ... collected from southern mainland China and Hainan Island.”: Which was caught where. Or in both? Clearly explain it.

**OUR REPLY**—From both. We had modified the manuscript following your comment.

--- end of comments and replies
