# Supplementary material for: Phylogeography of Aphyocypris normalis Nichols and Pope, 1927 at Hainan Island and adjacent areas based on mitochondrial DNA data
Source: PLoS One. 2023 Feb 28;18(2):e0282460. doi: 10.1371/journal.pone.0282460 (PMC9974131; doi:10.1371/journal.pone.0282460)
Supplement: Attachment — Submitted filename: Response to the reviewers R6-OK.docx [file pone.0282460.s009.docx]

**In response to Reviewer’s comments**

Author: I-Shiung Chen and Nian-Hong Jang-Liaw

Manuscript Title: Phylogeography of Aphyocypris normalis Nichols and Pope, 1927 at Hainan Island and adjacent areas based on mitochondrial DNA data (PONE-D-20- 37420R5)

Again, we deeply appreciate the reviewer’s great comments and suggestions on this work. However, we were surprised to find that the comments of the first reviewer (#4 reviewer) were based on the original version of our manuscript, which was submitted to PLOS ONE at the end of 2020, and has gone through 5 rounds of review so far. We appreciate the valuable comments provided by the #4 reviewer, nevertheless, we are unable to reply his comments for which are not suitable to discuss our latest submitted version.

The response to another reviewer’s comments are listed below:

Reviewer #5: The authors of the manuscript entitled Phylogeography of Aphyocypris normalis Nichols and Pope, 1927 at Hainan Island and adjacent areas based on mitochondrial DNA data sequenced mtDNA from 127 specimens and created a phylogenetic tree. The results indicated 3 major clades- one with wide geographic distribution and 2 that were more limited, however no obvious geographic barriers exist between them. There also appeared to be 8 biogeographic regions of A. normalis that correspond to climate. Based on results of the phylogenetic tree, The authors conclude that A. normalis diverged between 1.05-0.16 Ma, before the temporary land bridge existed across the Qiongzhou Strait.

Line 184 - can you please clarify- were the separate runs combined? Did they converge?

OUR REPLY— In this study MrBayes ran two completely independent and simultaneous analyses from different random trees. We set Nruns=2 to run the program. In the beginning of analysis, the two runs sampled different trees, but then they went convergence, the samples of the two trees were very similar. The trees of two runs converge at last.

Line 207- can you provide more information about parameters of the model were selected- tree prior, clock model, etc. Was any model selection performed? Are you using a model that was reported previously.

OUR REPLY— We had provided such information in Line 207-214 already.

Line 212- please check the references throughout. This reference was not correct.
OUR REPLY— We had checked the reference ([58] in the last version of manuscript) and corrected the references following your suggestion.

Line 544 subclade Ib AND Subclade III
OUR REPLY—We had corrected it following your suggestion.

Line 554 BY geographic barriers

OUR REPLY—We had corrected it following your suggestion.

Line 557-577- how do your phylogeographic regions differ by the ones described by He and Zhang.

OUR REPLY—We added a description in lines 514-517 to response your suggestion.

Line 605 - can you suggest any alternative explanations?

OUR REPLY—We had thought about that Clade III might be a lineage close to population in Indochina Peninsula, across South China Sea. However, we need additional specimen from Indochina Peninsula (Vietnam) to prove it. Currently, we only can discuss it within Hainan Island based our limited data.

Line 637 - and BECOME isolated

OUR REPLY—We had corrected it following your suggestion.

--- end of comments
