# Supplementary material for: Phylogeography of Aphyocypris normalis Nichols and Pope, 1927 at Hainan Island and adjacent areas based on mitochondrial DNA data
Source: PLoS One. 2023 Feb 28;18(2):e0282460. doi: 10.1371/journal.pone.0282460 (PMC9974131; doi:10.1371/journal.pone.0282460)
Supplement: Attachment — Submitted filename: Response to the reviewers R7.docx [file pone.0282460.s010.docx]

**In response to Reviewer’s comments**

Author: I-Shiung Chen and Nian-Hong Jang-Liaw

Manuscript Title: Phylogeography of Aphyocypris normalis Nichols and Pope, 1927 at Hainan Island and adjacent areas based on mitochondrial DNA data (PONE-D-20-37420R6)

Again, we deeply appreciate the reviewer’s great comments and suggestions on this work. The manuscript has been major revised accordingly as possible we can, and it had been modified completely almost. We accept almost all suggestions the reviewers offered. The following are our answers to the comments which we did not follow completely. Others were all accept by the authors.

**Reviewer #4**: In general I think the authors present a well-sampled dataset that provides additional knowledge of population structuring of this freshwater species. Below I provide two sets of comments. I do have two general concerns both revolve around the fact that this is a single-marker mitochondrial dataset. With that, I'd like to see more discussion on the limitations of such a dataset (i.e., it's not appropriate to look for gene flow using this type of data), and a bit more caution with how "deep" into the methods and analyses the authors go. I think restricting analysis to patterns of differentiation, haplotype patterns, dated phylogeny, etc., is appropriate. Inferring gene flow, or lack thereof with a single marker mitochondrial dataset is difficult if not inappropriate. Further, some discussion on how mitochondrial capture or sweeps could have affected the conclusions would be useful.

OUR REPLY—Thank you for your comments. Indeed multiple-markers datasets are more recommended on phylogeography researches currently, especially with nuclear DNA datasets. However, phylogenetic research using multigene datasets requires more funding/resource to perform as well. With limited financial support in this study, we must allocated resources carefully. Since one of our main goals is to analyze as many *A. normalis* as possible from different populations, our funding can only choose to use one marker for analysis. Based on our previous experience, nuclear genes always show fewer information on genetic variation rather than mtDNA markers. Under the condition of limited resources, we decided to use mtDNA *cytb* gene, a widely used marker on phylogenetic researches, in this study.

>Line 116: Does "tissue" mean frozen in this example? Or were these also ethanol-fixed, if so, you could just say "utilized ethanol-fixed tissue samples from...." if not, i'd change to "frozen and ethanol-fixed tissue samples from...."

OUR REPLY—The specimens we used should be "utilized ethanol-fixed tissue samples”. We had modified the manuscript following your comment.

>Line 403: Fst...need to clearly define in methods how this was calculated as there are multiple different ways to calculate Fst

OUR REPLY—Both AMOVA and Fst were computed using ARLEQUIN in this work. We had modified the manuscript following your comment.

>Line 407: I'm having a tough time with this interpretation, there are major issues with using a single mitochondrial marker to infer anything about geneflow...the mitochondrion doesn't recombine... Further, low genetic divergence between regions in general can be explained by just low divergence (neutral or selective) or gene flow. And when talking about a mitochondrial marker....gene flow would either produce no change or entirely erase an evolutionary history due to the fact that these regions don't recombine...they either don't interact or replace.
OUR REPLY—In theory you are right. However, mtDNA has been widely used in many phylogenetic studies and constructed many evolutionary hypotheses. We cannot fully understand all the details of the mtDNA mutation process, but the phylogenetic relationship trees constructed by mtDNA still has a certain reference value in systematic biology.

>Line 434: this is a poor definition of Fst, need to include at least some mention of relative...

OUR REPLY—We had modified the manuscript following your comment in Line 434—436.

>Line 473: insufficient isolation barrier or just recent isolation...without sufficient time to build up differential mutations even if completely isolated.

OUR REPLY—We think that the Qiongzhou strait plays an insufficient isolation barrier due to the short distance between Hainan and mainland; and the sea is shallow there. It is much possible to form a landbridge during the ice age in Qiongzhou strait.

>Line 574: I think adding some clarity and discussion here would be good. First, a landbridge only provide connectivity for terrestrial animals...it may be a stronger barrier for freshwater species if there are not connecting drainages than saltwater may be. I think some commentary on this should be made...given unknown connectivity of freshwater drainage systems between mainland china and hainan island during terrestrial continuity, we are uncertain if there was freshwater connection or not, and if not, this terrestrial barrier may have been stronger than a saltwater aquatic barrier....

OUR REPLY—We partially agree your point. However, the landbridge hypo-theses are in fact widely accepted in distribution researches on freshwater animals, such in those researches from Japan and Taiwan.

>Line 642: replace variation with divergence...however, as eluded to above...I'd restrain from making too strong claims on taxonomy and species status based on a single mitochondrial marker. Mitochondrial capture/sweeps could have occurred, erasing the mitochondrial history even with very limited gene flow, and the nuclear genome could retain very large differences, not detectable with cytb sequencing along

OUR REPLY—In this work, we displayed the genetic divergence in mtDNA aspect. This is our main goals in this project. It must be different from researches focused on other (nuclear) markers or NGS data. They must be good stories, but currently we like to tell the mtDNA one.

>Line 644: this sentence doesn't make sense...yes mtDNA is maternal, but comparing maternal origin with phylogenies at the species level or higher seems to not be a valid comparison. Comparing mitochondrial data with nuclear genomic data may provide different conclusions, using mtDNA data at population or species levels should change much...

OUR REPLY—Sometimes some mito-nuclear discordance on phylogeny comparisons in species level can be found in animals where males have larger daily movement distances and home-range sizes than females. The limitation of female movement may initiate phylogenetic structure of the maternally inherited mtDNA, while increased male movement drives broader gene flow of the nuclear genome. MtDNA data shows its unique value different from nuclear data in such case. It can contribute in phylogenies at the species level indeed.

General comments:
>Gene-flow: As mentioned above, I have strong reservations with inference of gene-flow, or lack thereof, from a single mitochondrial marker (cytb), see above for reasons. For this reason, I think it's important to either reduce the amount of discussion on gene flow or include a cautionary section on why a single mitochondrial marker and mitochondria in general are not good for detecting gene flow. Bottomline, I think there needs to be much more circumspection with inferring population structure and gene-flow from a single mitochondrial marker.

OUR REPLY—We agree your point. We also had mentioned in Line 650—654 to remind readers again that this is a single-marker study.

>Landbridges: I'd appreciate more discussion on land bridges and how they affect freshwater aquatic organisms...as mentioned above, a landbridge between Hainan and China may have been a stronger barrier to gene flow than the current saltwater barrier, and without known patterns of freshwater drainages on this landbirdge (I assume), it's difficult to accurately infer the expectation for degree of this barrier to a freshwater species.

OUR REPLY—It must be interesting to discuss the patterns of the possible freshwater drainages on landbirdge between Hainan and mainland. However, we found the references are very limited on the geology/oceanography in Qiongzhou strait, even from Chinese journals. We are sorry that such discussion is somewhat beyond the core objectives of this work (and our ability)…

**Reviewer #6**: The manuscipt of phylogeography of Aphyocypris normalis at Hainan Island present a rather interesting study, that after these 5 rounds of revision has been considerably improved. There are only a few minor points that, in my opinion need revision, abd thus I suggest the manuscript to be accepted right after the authors proceed with these corrections, without the need for a further revision round.

OUR REPLY—Thank you very much for your helpful comments and long-term contribution on this work. Thank you.

>Line 439: SAMOVA indeed showed that K=3 has the highest value in terms of grouping options; however, as K=4 was also very very close to this value, I would like the authors to say a few things more about this clustering option as well. What was this fourth cluster that exhbited such close possiblity?

OUR REPLY—Indeed, the Fct values of K=3 and K=4 are very close. The groupings of both hypothese are very close as well; only the Ninyuang R’s specimen was separated from others (populations of Changhua and Wanlou Rivers) in K=4 hypothesis. In our earlier version of the SAMOVA results we described both grouping hypotheses, but it seemed too details and we remove the K=4 hypothesis in our original manuscript. We remind the K=4 result briefly following your suggestion in this revised manuscript following your comment.

--- end of comments
