# Supplementary material for: Phylogeography of Aphyocypris normalis Nichols and Pope, 1927 at Hainan Island and adjacent areas based on mitochondrial DNA data
Source: PLoS One. 2023 Feb 28;18(2):e0282460. doi: 10.1371/journal.pone.0282460 (PMC9974131; doi:10.1371/journal.pone.0282460)
Supplement: Attachment — Submitted filename: Response to the reviewers R8.docx [file pone.0282460.s011.docx]

**In response to Reviewer’s comments**

Author: I-Shiung Chen and Nian-Hong Jang-Liaw

Manuscript Title: Phylogeography of Aphyocypris normalis Nichols and Pope, 1927 at Hainan Island and adjacent areas based on mitochondrial DNA data (PONE-D-20-37420R7)

Again, we deeply appreciate the reviewer’s great comments and suggestions on this work. The manuscript has been major revised accordingly as possible we can, and it had been modified completely almost. We accept almost all suggestions the reviewers offered. The following are our answers to the comments.

**Reviewer #7**: The article describes the values of genetic diversity, as well as its spatial distribution and ancestry-descent relationships based on a single gene. This is a limitation, but the information is undoubtedly relevant and provides a coherent explanation for the observed patterns. This limitation of using a single gene is identified by the authors, who suggest interesting lines of research proposals for the future.


>>212 I suggest the use of mode of previously reported molecular substitution rate instead mean

OUR REPLY—Thank you for your suggestion. The previously reported molecular substitution rates for cyprinid cytb ranged from roughly 0.6% to 1.3% Myr. We had adopted a frequently-used cyt*b* clock rate covered in above range, following Ketmaier et al. [63] and Zhang et al. [64]. Please allow us to keep the former selection in the manuscript.

>>234 Combining the 19-25 populations that have intermediate to the Songtao Reservoir seems like a good idea, but can't a lentic system be a barrier for organisms that inhabit lotic systems? Explain

OUR REPLY—It is a good idea to discuss the barrier mechanism of Songtao Reservoir on freshwater animals in Hainan. A huge lentic system such as Songtao Reservoir can be a barrier for small-sized non- pelagic freshwater fish like *Aphyocypris normalis*. It is too difficult and risky for adult *A. normalis* to move through a deep, wide water body, for they will be easy to be hunted by predators during their movement without hidden substrates they prefer. Pelagic phase eggs or larvae are possible, but with huge risk for being food of others as well. Populations of sites 19—25 were widely separated by Songtao Reservoir in Nandu River as two parts (sites 19—22 in the upstream of Songtao Reservoir, and sites 23—25 in the downstream of it), and the genetic data were I accordance with this distribution pattern.

We had modified the manuscript following your comment in lines 547—559.

>>241-242 Define what values of Fst are considered High and low and references if corresponds ej Freeland 2005

OUR REPLY—We had modified the manuscript following your comment in lines 440—442 and added the reference Freeland 2018 as [72].

>>243 Mention if the Fst values were computed using the information of sequences or just the haplotype frequency as the reviewer 4 suggested previously

OUR REPLY—The Fst values were computed using the information of haplotype instead of all sequences. We had modified the manuscript following your comment in lines 243—244.

>>272 Include the transition transversion ratio and mention the number of private haplotypes

OUR REPLY—The estimated Transition/Transversion ratio is 11.50, which was conducted by MEGA, and 41 are private haplotypes. We had modified the manuscript following your comment in lines 272—275.

>>278-283 It would be a good idea to explain in the discussion that the pattern of lower diversity in islands is common with respect to the continent and can be used to approximate the historical Ne of the populations. It can be suggested that in Zhubi river there is a higher Ne, i.e. how can it be explained that with an N of 2 it is the highest value? Considering that there are sites such as Wanquan river with n =10 with zero h and pi

OUR REPLY—Thank you for your nice suggestion. We had modified the manuscript following your comment in lines 493—502.

>>285. Explain the average number of differences between these 12 haplotypes to get an idea of whether they are single nucleotide substitutions or involve several substitutions.

OUR REPLY—These haplotypes could be separated into two groups. One belonged to Clade I (h4, h6, h26, h28, and h29), in which they were single or two nucleotide substitutions to each other. Another belonged to Subclade Ib (h20, h21, h22, h23, h24, h25, and h27), in which they are single or several substitutions (up to 11) to each other. We had modified the manuscript following your comment in lines 286-290.

>>286-287 Indicate the number of differences of the shared haplotype between the Wanquan and Nandu, with respect to their closest haplotype. I.e. both systems are close but it is possible that it is the same haplotype by convergence?

OUR REPLY—The haplotypes found in Nandu R. were h4, h6, and h20—h29 (please check the Table 1), and the only haplotype found in Wanquan R. were h4. According to h4 could be found in other river system (Xi R. of Pearl R.in mainland, in our opinion, the h4 is possibly the most ancestral haplotype in this study, for its wide distribution and central position in the statistical parsimony haplotype network (Fig.4), rather than being a convergence haplotype in different river systems.

>>292 haplotype 4 the ancestral one? Explain
>>366-369 h4 and h1 are the ancestral ones? Explain

OUR REPLY—Both h4 and h1 are the possible ancestral ones. They were present in more than three populations, and h4 is the most frequent by far. Besides, h4 is also the only haplotype shared between Hainan Island and mainland China (sites 4, 5, 23, A5; Fig. 1). These are the characteristics of possible ancestral haplotypes, and h4 is more possible to be ancestral haplotype than h1 for a wider distribution across the strait. In Fig. 4 the h4 occupies the center of the statistical parsimony haplotype network. It is also a characteristic of possible ancestral haplotypes.

>>419-420 Are there data on the characteristics of the rivers? Oxygen, temperature, which may be more relevant than what happens out of the water?

OUR REPLY—We have no data on the characteristics of the rivers. It is somewhat pity to absent from it in discussion. However, please understand that it will be too complex to have such discussion in this paper.

>>475-479 Mention that in addition to isolation by distance, there are other alternatives (e.g. isolation by environment).

OUR REPLY—We had modified the manuscript following your comment in lines 592-593.

>>491-496 Since the strait is not so relevant is it worth considering the depth of the strait, that space was land at glacial maximum?

OUR REPLY—Based on our data and other’s works, the geographic isolation of the Qiongzhou strait between Hainan Island and mainland China did not create efficient isolation barriers. However, it is necessary to point it out clearly for it is generally accepted that geographical isolation is a very efficient isolation mechanism for freshwater animals. Furthermore, a former reviewer suggested us to describe the depth information of Qiongzhou Strait. For some readers it seems being necessary to describe the depth of the strait anymore.

>>497-498 Water characteristics of lotic systems this may be more relevant than just precipitation or temperature.

OUR REPLY—We had modified the manuscript following your comment in Line 508.

>>S2 please change the order of the two below figures d and c

OUR REPLY—We think that you meant S1. We had modified the order of the two below figures d and c following your suggestion.
